# Diagnostic interval of inflammatory bowel disease in Chinese children and its relationship with growth parameters: a retrospective study

**DOI:** 10.3389/fped.2025.1465694

**Published:** 2025-02-05

**Authors:** Juan Zhou, BinRong Chen, ZhiCheng Wang, Li Liu, HongJuan OuYang, YanHong Luo, WenTing Zhang, ChenXi Liu, MeiZheng Zhan, JiaQi Duan, CanLin Li, Na Jiang, JieYu You, HongMei Zhao

**Affiliations:** ^1^ Department of Gastroenterology and Nutrition, The Affiliated Children’s Hospital of Xiangya School of Medicine, Central South University (Hunan Children’s Hospital), Changsha, Hunan, China; ^2^ The School of Pediatrics, Hengyang Medical School, University of South China (Hunan Children’s Hospital), Changsha, Hunan, China

**Keywords:** inflammatory bowel disease, children, delayed diagnosis, Crohn’s disease, Chinese

## Abstract

**Background:**

Delayed diagnosis of inflammatory bowel disease (IBD) is common in Europe and North America, with limited research in Asia. We aimed to investigate factors influencing delayed diagnosis of IBD in Chinese children and the impact of delayed diagnosis on growth.

**Methods:**

This was a retrospective study. Clinical data on children with IBD were collected through electronic medical records. The diagnostic interval includes the time from symptom onset to hospital admission and admission to diagnosis. Diagnostic delay was defined as the upper quartile of the time interval from the first symptom to the diagnosis of IBD. For the effect on growth indicators, the length of follow-up was at least 3 months from diagnosis.

**Results:**

This study included 222 children with IBD, predominantly with Crohn's disease (86.0%). Approximately one-quarter of children require more than 366 days to be diagnosed with IBD, primarily due to the extended interval between the onset of initial symptoms and hospital admission. Multivariate logistic regression models showed that fever was associated with a prolonged time interval from first symptom onset to admission and the odds ratio (OR) was 0.45 [95% confidence interval (CI) 0.22–0.94]. Age and bloody stools were associated with prolonged intervals from admission to diagnosis, with ORs of 0.84 (95% CI 0.77–0.92) and 0.36 (95% CI 0.14–0.94), respectively. Delayed diagnosis was associated with height at first admission and follow-up. Children with a delayed diagnosis had a 5.87-fold higher chance of growth retardation upon initial admission compared to children without a delayed diagnosis (95% CI 1.59–24.05). After 15.7 months of follow-up, this elevated risk remained (OR 3.28, 95% CI 1.00–10.50).

**Conclusion:**

Delayed diagnosis is common in Chinese children with IBD and is associated with persistent height impairment.

## 1 Introduction

Inflammatory bowel disease (IBD), encompassing ulcerative colitis (UC), Crohn's disease (CD), and unclassified cases (IBD-U), is a chronic gastrointestinal disorder characterized by relapses. It is most prevalent during adolescence and young adulthood, with a rising incidence in the pediatric population (1). Individuals with childhood-onset IBD exhibit a more severe phenotype, extensive intestinal inflammation, frequent flare-ups, and often experience impaired growth (2, 3). Research indicates that 65%–85% of children and adolescents diagnosed with CD experience stunting and impaired nutritional status, with 15%–40% experiencing ongoing growth deficits (4, 5). Furthermore, a delayed diagnosis can result in complications such as bowel strictures, fistulas, and an elevated risk of intestinal surgery (6). Therefore, prompt detection and diagnosis are crucial for improving outcomes in pediatric patients.

The clinical manifestations of IBD are varied, encompassing classic symptoms like weight loss, abdominal pain, and diarrhea, as well as non-classic symptoms, such as impaired growth and anemia. As the initial presentation of IBD is often not classical, diagnosing IBD can be challenging and prolonged. Studies have shown that a significant portion of patients with UC and CD are diagnosed after more than 7 and 15 months, respectively. Research in pediatric IBD has highlighted common delays in diagnosis, with a median delay of 2.0–10.4 months overall (range 2.0–18.0 months for UC and 4.0–24.0 months for CD) (7). Although most studies have focused on European and North American populations (7–9), limited evidence exists for Asian populations. Asian children exhibit a lower prevalence of IBD and different disease phenotypes compared to Caucasians (10), as evidenced by a higher perianal involvement at onset (11), suggesting that there may be potential differences in the diagnostic timeline between these groups. Moreover, research into specific factors contributing to the delayed diagnosis of IBD is lacking, necessitating further investigations to identify consistent factors (12, 13). Therefore, there is a critical need to explore the diagnostic interval of IBD in Asian children and its impact on growth.

A retrospective study was conducted at a provincial tertiary specialized hospital in China to investigate the time interval from symptom onset to IBD diagnosis. This included the time from symptom onset to admission and from admission to IBD diagnosis. Factors contributing to delayed diagnosis were also examined, along with the impact of delayed diagnosis on growth parameters.

## 2 Materials and methods

### 2.1 Study population

**Figure 1:**
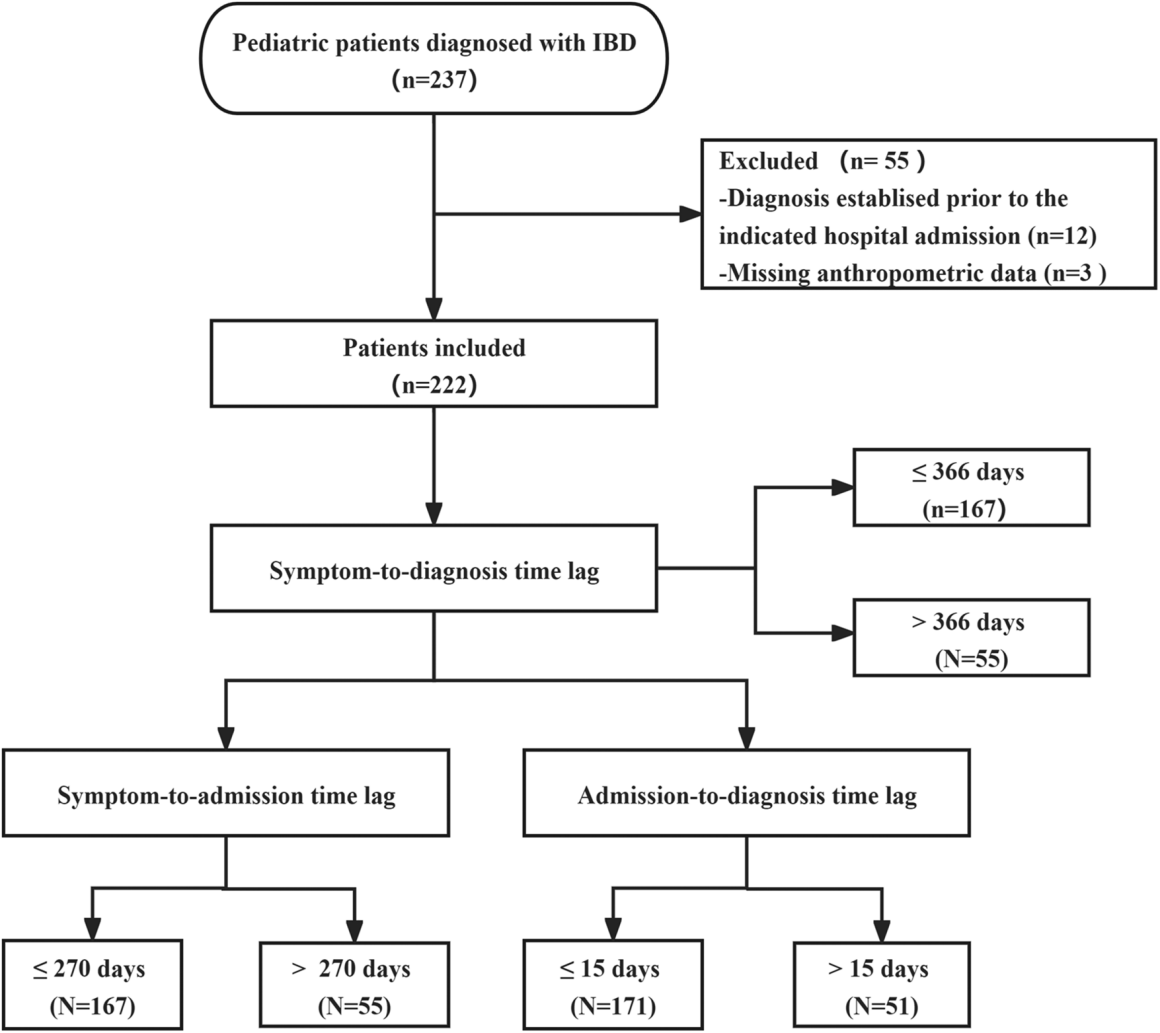
Flow diagram of study population.

This was a retrospective study that collected and analyzed electronic medical records of children with IBD diagnosed at Hunan Children's Hospital between 2017 and 2023. Figure 1 depicts the flow chart of the study population. A total of 237 patients with confirmed IBD were initially included, with 12 patients excluded for pre-existing IBD and 3 patients excluded due to insufficient information. This resulted in a final analysis of 222 patients. The study was approved by the Ethics Committee of Hunan Children's Hospital (approval number: HCHLL-2022-154) and the requirement for informed consent was waived.

### 2.2 Data collection

The data of children with IBD were collected through electronic medical records, including demographic characteristics (sex, age), symptoms and signs (diarrhea, bloody stools, abdominal pain, fever, vomiting, perianal symptoms, skin manifestations, oral ulcers, arthritis, reported weight loss, reported linear growth impairment), and relevant dates (date of symptom onset, date of admission, date of IBD diagnosis, and date of follow-up). In addition, laboratory parameters, such as C-reactive protein (CRP), erythrocyte sedimentation rate (ESR), and hemoglobin, as well as growth parameters such as body mass index for age Z-score (BAZ) and height for age Z-score (HAZ), were included. Before data collection, all parameters were extracted from medical records by trained data collectors. To ensure uniform data collection and accuracy, all variables were defined before data extraction and placed in a standardized format during data collection. Once data collection was complete, two additional (independent) data abstractors verified the accuracy of all records and compared all data with the electronic medical record.

### 2.3 Definition

Following the Expert Consensus on the Diagnosis and Treatment of Inflammatory Bowel Disease in Children (14), the diagnosis of IBD involves a comprehensive assessment of clinical symptoms, endoscopy, histopathology, and imaging. The diagnosis was in accordance with the guidelines of the European Society for Pediatric Gastroenterology, Hepatology, and Nutrition (revised Porto criteria) (15). Features of CD include the following: (1) recurrent episodes of abdominal pain with weight loss and growth failure, and other possible symptoms or signs such as diarrhea, fever, and perianal lesions; (2) endoscopy shows the segmental and asymmetric distribution of the lesions, and typical CD manifestations (e.g., aphthous ulcers, paving-stone-like changes, and jumping lesions) can be seen; (3) biopsy histology reveals patchy cryptitis, crypt abscesses, ileitis, or pathognomonic non-caseating granulomas; and (4) imaging suggests rigid stenotic segments, skip areas, and sinus tracts or fistulas. Features of UC include the following: (1) persistent bloody stools with diarrhea; (2) endoscopy showing lesions starting in the distal rectum and seeing continuous, diffuse mucosal inflammation; and (3) biopsy histology showing structural changes in the crypts and an inflammatory infiltrate. IBD-U was defined as a lesion confined to the colon, and it was not possible to differentiate between CD and UC. Anemia was defined based on the diagnostic criteria established by the World Health Organization. The diagnostic interval, which signifies the time from initial symptom onset to the diagnosis of IBD, was further analyzed by dividing it into two periods: the time from symptom onset to hospital admission, and the time from admission to diagnosis. In keeping with previous studies (8, 16, 17), diagnostic delay was identified as the upper quartile of the time taken from symptom onset to the diagnosis of IBD. Similarly, prolonged symptom-to-admission and admission-to-diagnosis intervals were defined as the upper quartile of their respective periods.

### 2.4 Statistical analysis

The continuous variables were expressed using median and interquartile range (IQR) and the categorical variables were expressed in frequencies (proportions). Logistic regression analyses were conducted to determine the odds ratio (OR) and 95% confidence interval (CI) for assessing associations. Multivariable models were developed using Wald backward selection with variables from univariate analysis having a *P*-value <0.2. All statistical analyses were performed using R software version 4.1.2. A *P*-value <0.05 was considered statistically significant.

## 3 Results

**Figure 2:**
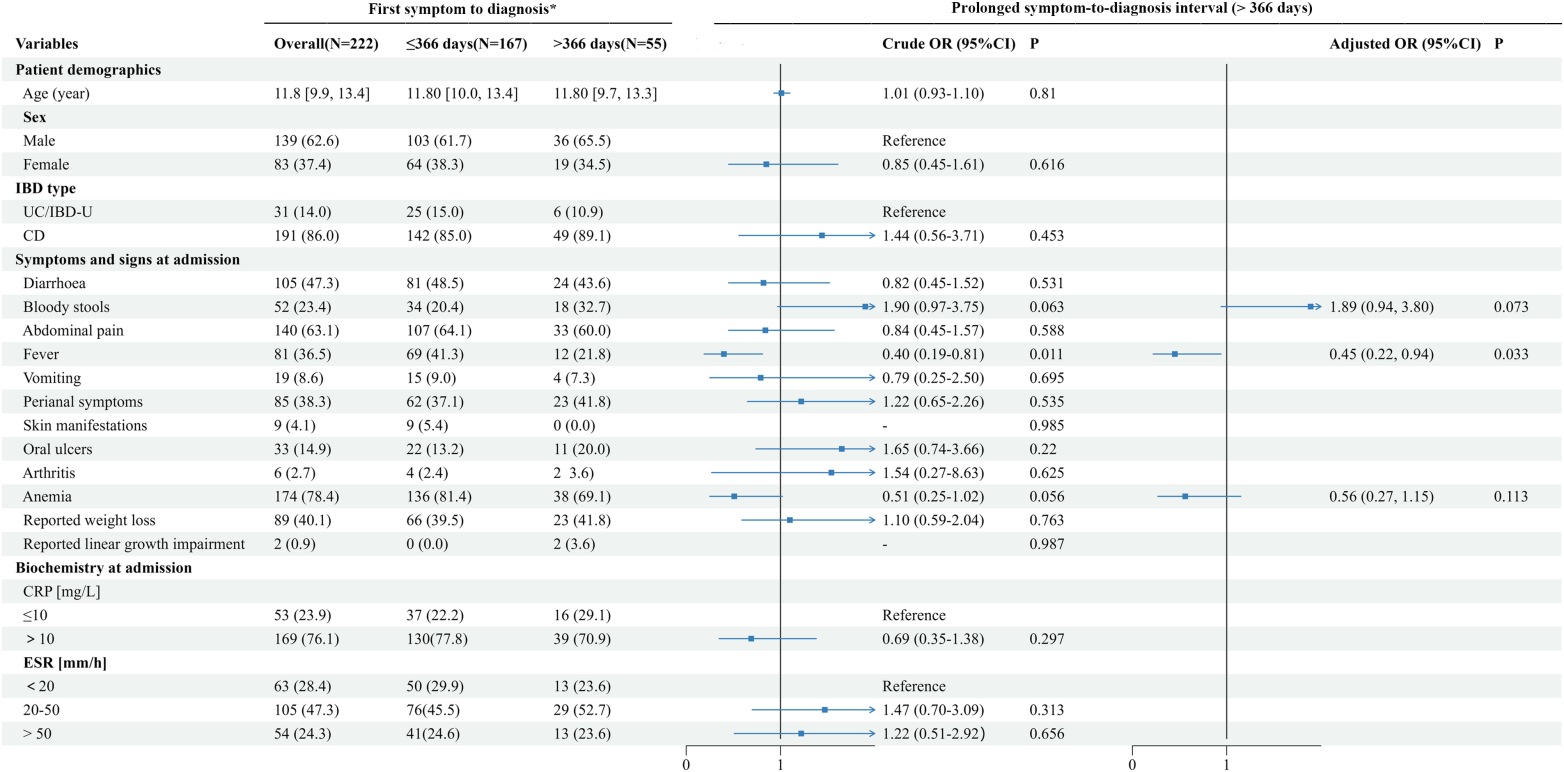
Factors associated with prolonged symptom-to-diagnosis interval (>366 days) in children with IBD. *Data are expressed as the median (interquartile range) for continuous variables or counts (percentages) for categorical variables. Multivariate models were constructed using Wald backward selection for variables with *P* < 0.2 in univariate analyses. CD, Crohn's disease; CI, confidence interval; CRP, C-reactive protein; ESR, erythrocyte sedimentation rate; OR, odds ratio; IBD-U, IBD-unclassified; UC, ulcerative colitis.

The characteristics of pediatric patients with IBD classified by time lag categories are detailed in Figure 2. The patients were mostly boys (62.6%) with a median age of 11.8 years (IQR 9.9–13.4). The vast majority (86.0%) of children were diagnosed with CD, while only 31 (14.0%) were diagnosed with UC or IBD-U.

Children with IBD were admitted to the hospital with a variety of clinical manifestations, with abdominal pain, diarrhea, weight loss, perianal manifestations, fever, blood in the stool, and oral ulcers predominating. In comparison, fewer children had vomiting, skin manifestations, arthritis, and linear growth impairment. The percentage of children with anemia was as high as 78.4% (Figure 2).

From the onset of symptoms to the diagnosis of IBD, the median period was 117.0 days (IQR 41.0–366.0). In total, 55 (24.8%) patients were diagnosed after 366 days. The median time from the first symptom onset to hospital admission was 75.0 days (IQR 30.0–270.0), and the median time from hospital admission to IBD diagnosis was 10.0 days (IQR 7.0–15.0).

Figure 2 shows the factors associated with a prolonged symptom-to-diagnosis interval (>366 days). It was found that fever was negatively correlated with a prolonged symptom-to-admission interval (Supplementary Figure 1), as well as the prolonged symptom-to-diagnosis interval (Figure 2). The adjusted ORs for these associations were 0.45 (95% CI 0.22–0.88) and 0.45 (95% CI 0.22–0.94), respectively (Supplementary Figures 1, 2). Upon admission, older age and the presence of bloody stools were found to be correlated with a quicker diagnosis of IBD, with adjusted ORs of 0.84 (95% CI 0.77–0.92) and 0.36 (95% CI 0.14–0.94), respectively (Supplementary Figure 2).

The impact of delayed diagnosis on growth was further investigated by excluding 40 children with IBD who did not receive follow-up or were followed up for less than 3 months. A total of 182 children with IBD were followed for a median duration of 15.7 months (IQR 8–21).

**Figure 3:**
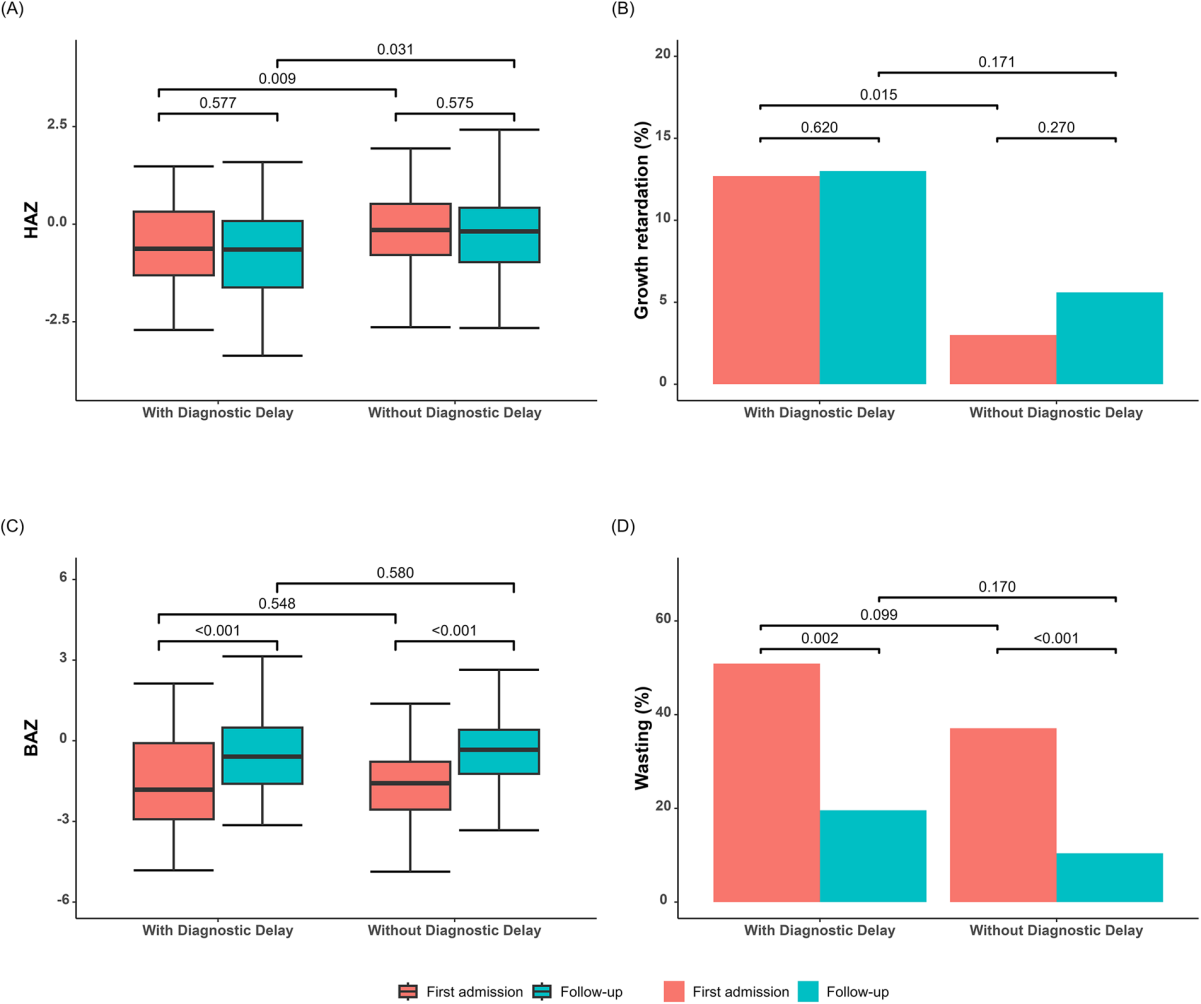
Growth parameters of children with and without diagnostic delay. (**A**) HAZ at first admission and follow-up in both groups. (**B**) Proportion of children with growth retardation at first admission and follow-up in both groups. (**C**) BAZ at first admission and at follow-up in both groups. (**D**) Proportion of wasting at first admission and at follow-up in both groups. BAZ, body mass index for age Z-score; HAZ, height-for-age Z-score.

At first admission, approximately 5.6% of the children exhibited growth retardation and 38.5% showed wasting. Children with a delayed diagnosis had significantly lower HAZ compared to those without a delayed diagnosis (Figure 3A), while BAZ did not show a significant difference (Figure 3C). After a follow-up period of 15.7 months, there was no significant change in HAZ but a notable increase in BAZ. The HAZ of children with a delayed diagnosis remained lower than those without a delayed diagnosis during the follow-up period.

The logistic regression results revealed that children with a delayed diagnosis had a 5.87 (95% CI 1.59–24.05) times higher likelihood of experiencing growth retardation upon first admission compared to children without a delayed diagnosis (Table 1). This increased risk persisted even after a 15.7-month follow-up period (OR 3.28, 95% CI 1.00–10.50). Notably, delayed diagnosis did not show any significant association with wasting, with ORs of 1.34 (95% CI 0.66–2.71) and 2.20 (95% CI 0.82–5.59), respectively.

**Table 1:** The association between delayed diagnosis and growth parameters.

	Growth retardation		Wasting	
Time	OR (95% CI)	*P*	OR (95% CI)	*P*
First admission	5.87 (1.59–24.05)	0.008	1.34 (0.66–2.71)	0.417
Follow-up	3.28 (1.00–10.50)	0.043	2.20 (0.82–5.59)	0.104

## 4 Discussion

This study of Chinese children found that approximately one-quarter of children with IBD were diagnosed after more than 366 days. The time to diagnosis was even shorter if the child had a fever. Older age and the presence of bloody stools were diagnosed more quickly upon hospital admission. In addition, delayed diagnosis was associated with persistent height impairment, and the association with wasting was not found.

Similar to the present study, CD predominates among children with IBD in Singapore, and the incidence of CD is approximately double that of UC in 2013–2015 (18). There is a continued increase in the incidence of pediatric CD and UC globally. Summarizing epidemiological data from Asian populations, Huang and Aw found a steep rise in the incidence of CD in children and a gradual narrowing of the UC-CD incidence ratio to a point of inversion of the ratio (11). In addition, this study discovered that a greater number of boys were affected compared to girls. Population-based studies of the last two decades have shown that adult CD exhibits a slight female predominance in Western countries, but not in Asia (19). Kim et al. described the clinical characteristics of 594 Korean children with CD and showed that 70.9% of the patients were boys (20). A large meta-analysis by Shi et al. of 525,425 (adult and pediatric) patients with IBD similarly showed that CD in Asians, but no other ethnicities, displayed male dominance (21). There is no explanation for the difference in gender distribution of Asian patients with CD, which may be related to genes and environmental exposures. Further studies are needed in the future to elucidate the role of gender as a disease modifier in IBD.

According to the findings of this investigation, prompt diagnosis of IBD remains problematic. The median diagnostic interval from the first onset of IBD-related symptoms to IBD diagnosis was 117 days, and approximately one-quarter of children with IBD were diagnosed after more than 1 year. The data are similar to those from earlier pediatric research, with Arcos-Machancoses et al. (22) reporting a median diagnostic interval of 84 days, Timmer et al. (23) of 120 days, and Sulkanem et al. (24) of 150 days. Interestingly, the time to diagnosis was slightly longer in some older studies, at approximately 210–329 days, perhaps reflecting continued advances in IBD diagnostic modalities and medical care (25, 26). However, in some of the recent studies conducted in West and South Asia, the time to diagnosis was considerably longer, approximately 180–360 days, suggesting significant country-related disparities (27, 28).

Consistent with the results of studies conducted in Finland and Saudi Arabia, the present study showed that the time interval between symptom onset and hospital admission was significantly higher than the time interval between admission and IBD diagnosis (24, 27). In contrast, results from adult studies have shown longer intervals between admission and final diagnosis (13, 29) which may be related to children's inability to clearly describe their symptoms and insufficient parental attention. In addition, early signs and symptoms of IBD are often inconspicuous, non-specific, and identical to those of other pediatric digestive disorders, and thus IBD may be overlooked by family members.

The comparison between the delayed and non-delayed groups revealed several important findings. First, patients in the non-delayed group exhibited a significantly higher frequency of fever compared to those in the delayed group. This aligns with the findings from a Chinese cohort study on adult patients with IBD (30), indicating that fever is a key symptom of IBD of which physicians should take note. Second, the presence of blood in the stool aids in the early diagnosis of children after hospital admission. Studies by Ricciuto et al. (9) and Sawczenko and Sandhu (31) showed that bleeding or bloody stools are linked to a shorter delay in diagnosing IBD in children. Moreover, Ricciuto et al. (9) found that diarrhea was associated with a shorter delay, whereas Spray et al. (26) reported that symptoms other than diarrhea were linked to a longer delay. The discrepancies in results across studies may stem from variations in study populations and definitions of clinical characteristics. The young age of the child could result in a delay between admission and diagnosis, possibly due to the gradual emergence of bowel disease symptoms and the reluctance to use invasive treatments on young children. In addition, cases of IBD in patients aged under 6 years (referred to as very early onset IBD) are uncommon, leading clinicians to initially explore other potential diagnoses before considering IBD, such as allergic colitis or protracted gastroenteritis.

As previously reported, children with IBD have poor nutritional status. On admission, approximately 5.6% of the children showed growth retardation, while 38.5% exhibited wasting. After 15.7 months of treatment, wasting had greatly decreased, but growth retardation remained unchanged. As weight is influenced by several factors, weight-for-age is seen as a short-term indicator of inadequate dietary intake or nutrient utilization, which can lead to a long-term decline in height-for-age (32). With timely adjustment and treatment, weight can usually be regained relatively quickly, while height often cannot. In the present study, delayed diagnosis was found to be associated with persistent height impairment. Ricciuto et al. (8) found an independent association between delayed diagnosis and growth impairment. The longer the delay in diagnosing IBD, the lower the HAZ value, suggesting that a delay in the diagnosis of IBD has a serious impact on children's height. A single-center study conducted in Canada also noted that impaired height due to delayed diagnosis is difficult to recover from within a short period, and this persists 1 year after diagnosis (9). Growth failure is caused by a combination of factors, including undernutrition itself, the inflammatory process, and pubertal delay. IBD is characterized by widespread intestinal inflammation, which may be an important mediator of growth disorders in children with IBD. Inflammation inhibits insulin-like growth factors 1 through pro-inflammatory cytokines, such as tumor necrosis factor α and interleukin 6, which in turn affects growth (33). In addition, children with IBD often experience hypogonadism and delayed puberty, which results in slower height growth and may not experience full catch-up growth before the epiphyses close (34). Of course, a delayed diagnosis is associated with persistent height disorders with IBD, mainly due to the lack of timely nutritional support and treatment. A timely diagnosis is of great significance for improving the growth of children with IBD.

Our study contains some merits. First, the use of Chinese children as the study population contributed to the expansion of data on IBD. Second, this study divided the time to diagnosis into two parts: time from symptom onset to admission and time from admission to diagnosis, to demonstrate that the time from symptom onset to admission is the primary cause of the delayed time to diagnosis. Third, the segmentation looked at the factors that may be associated with a delayed diagnosis of IBD in children to offer a reference point for prompt IBD diagnosis. Finally, this study looked into the relationship between delayed diagnosis and growth indicators, emphasizing the consequences of delayed diagnosis in children.

The present study has some limitations. First, this is a single-center study and failed to cover children from different regions; therefore, it may not accurately reflect the diagnostic time interval and the nutritional status of children in China. Second, considering that there may be differences in medical resources and services in different regions, it may also be difficult to discover the factors related to delayed diagnoses in other regions. Conducting a multicenter study will likely reveal more comprehensively the factors related to a delayed diagnosis of IBD. Third, this is a retrospective study and the data were obtained by collecting electronic clinical data. The time of symptom onset is based on the recollection of the child or family members and may be subject to information errors, such as recall bias. However, during the data collection process, we adopted standardized methods and performed data checks to minimize errors. We chose to study children who were diagnosed with IBD for the first time. Many children fail to receive satisfactory treatment or are misdiagnosed at lower hospitals, leading to a further prolongation of diagnosis and further delays. The validation of the fact that the time between symptom onset and referral to a pediatric gastroenterologist is the largest factor affecting the diagnostic time of IBD in children further emphasizes the need to enhance the awareness of IBD among primary care physicians, including community physicians. We advocate the provision of up-to-date IBD teaching to primary care physicians through the Digital Village System (35) and establish effective ways for primary care hospitals to communicate with specialized hospitals. In addition, clinicians need state-of-the-art knowledge of IBD to be able to administer treatment safely and effectively even before a full diagnosis has been completed and to reduce the harm caused by a delayed diagnosis. Families of children with IBD also need to increase their awareness of the need for timely access to medical care and grasp the key points of diagnosis to shorten the diagnostic time of children with IBD.

A delayed diagnosis is common in Chinese pediatric patients with IBD, which is associated with persistent height impairment. The time interval between symptom onset and hospital admission contributes the most to the overall diagnosis time. This is critical for future intervention strategies for pediatric IBD. Increased awareness of IBD among public and primary care physicians is necessary to reduce diagnostic delays in pediatric IBD.

## Data Availability

The original contributions presented in the study are included in the article/Supplementary Material, further inquiries can be directed to the corresponding author.

## Supplementary material

Supplementary Figure 1 Factors associated with prolonged symptom-to-admission interval (>270 days) in children with IBD. *Data are expressed as the median (interquartile range) for continuous variables or counts (percentages) for categorical variables. Multivariate models were constructed using Wald backward selection for variables with *P* < 0.2 in univariate analyses. CD, Crohn's disease; CI, confidence interval; CRP, C-reactive protein; ESR, erythrocyte sedimentation rate; OR, odds ratio; IBD-U, IBD-unclassified; UC, ulcerative colitis.

Supplementary Figure 2 Factors associated with prolonged admission-to-diagnosis interval (>15 days) in children with IBD. *Data are expressed as the median (interquartile range) for continuous variables or counts (percentages) for categorical variables. Multivariate models were constructed using Wald backward selection for variables with *P* < 0.2 in univariate analyses. CD, Crohn's disease; CI, confidence interval; CRP, C-reactive protein; ESR, erythrocyte sedimentation rate; OR, odds ratio; IBD-U, IBD-unclassified; UC, ulcerative colitis.
