# Supplementary figures and images for: Diagnostic interval of inflammatory bowel disease in Chinese children and its relationship with growth parameters: a retrospective study
Source: Front Pediatr. 2025 Feb 5;13:1465694. doi: 10.3389/fped.2025.1465694 (PMC11835845; doi:10.3389/fped.2025.1465694)

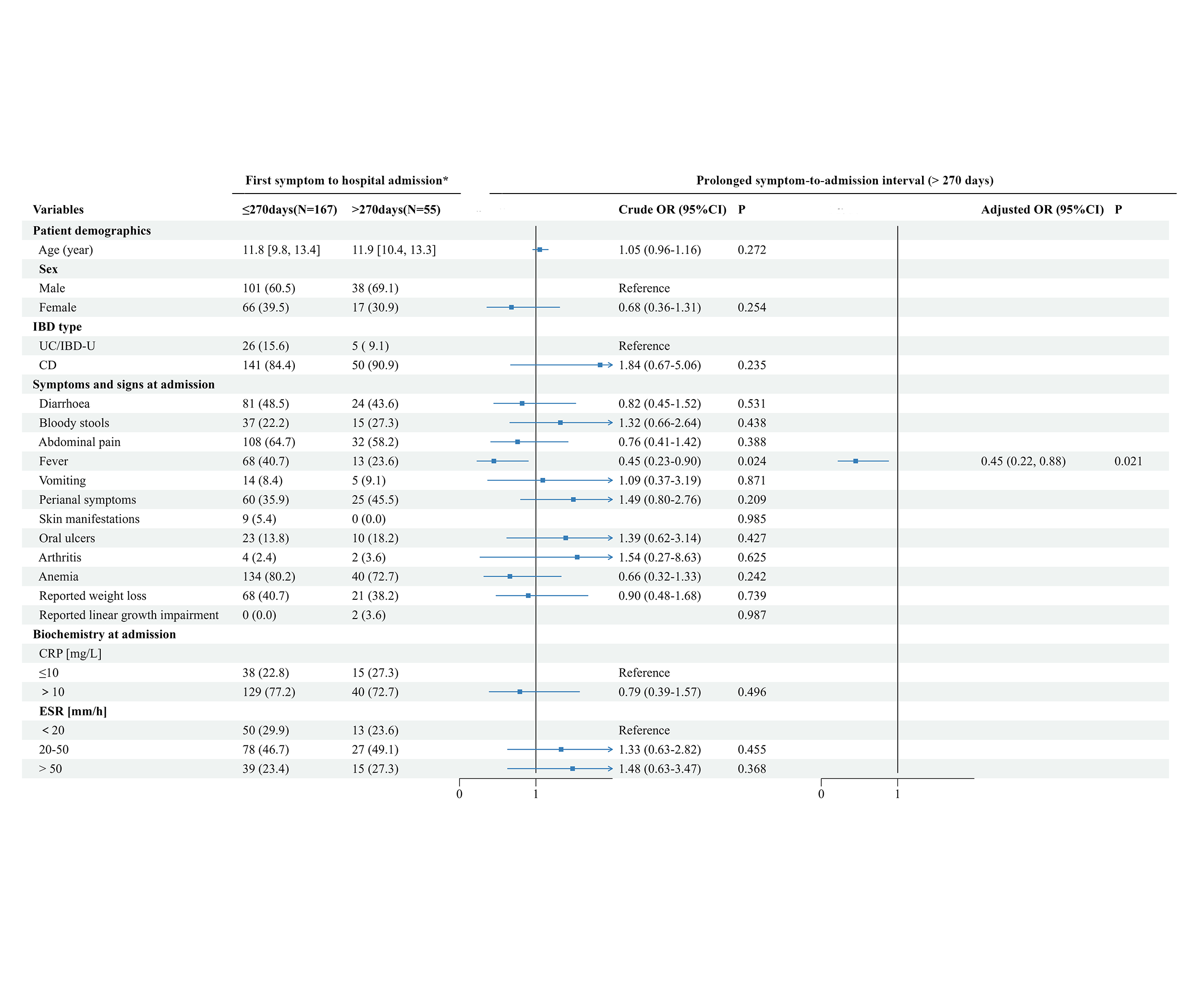

Supplement: Supplementary Figure 2 — Factors associated with prolonged admission-to-diagnosis interval (>15 days) in children with IBD. *Data are expressed as the median (interquartile range) for continuous variables or counts (percentages) for categorical variables. Multivariate models were constructed using Wald backward selection for variables with P < 0.2 in univariate analyses. CD, Crohn's disease; CI, confidence interval; CRP, C-reactive protein; ESR, erythrocyte sedimentation rate; OR, odds ratio; IBD-U, IBD-unclassified; UC, ulcerative colitis. [file Image1.tif]
